# Revival in Dental Journalism

**Published:** 1874-05

**Authors:** S. E. Harryman

**Affiliations:** Lawrenceburg Ind.


### REVIVAL IN DENTAL JOURNALISM.

LAWRENCEBURG IND., March 23, 1874.

TO THE EDITOR OF THE DENTAL REGISTER :

In looking over the library of my worthy, preceptor, (one of the oldest dentists in years of practice in the country) I am always deeply interested in the old journals found there, they are full of that which is spicy, practical and of good common sense, and, so far as the profession was then developed, were doubtless fully up to the times.

I have often thought that those journals, exhibited more genuine devotion to the interests of the dentist, and his patients, than the professional literature of to-day, or, I had better say, some days ago, for I think we have decidedly improved in that line during the last year or two.

Great credit is due the noble men who are aiding the profession by their inventions and discoveries.

Greater, yet, is due those who faithfully bring before the profession the merits of those discoveries and inventions, and keep us who have not the oportunity to partake, informed of the proceedings of distant associations of our brethren. Why ! its almost as good as being there to take up a journal and read of all that is done, and then the tedious preliminaries are abreviated, or omitted, and a fellow draws a sigh of relief, to think he has escaped being bored with that. It is human nature, Mr. Editor, to desire the kernel that somebody else has kindly picked out of the shell, and not have the trouble of working through the outside crust ourselves. But I do not think we are in danger of being injured by an abundance of information. My own experience is, that not one day's practice passess without my having great cause of gratitude to one or more of a score of our professional benefactors, and instead of feeling a lack of ambition, because of the ease of obtaining and using the ideas, and experience of others, I feel spurred on to do my part, that I may be found worthy to use these privileges, if not to emulate the originators.

Now I might go on for a page or two, but will not intrude on your valuable time, considering that enough has been said to convince you, that one at least, has been stirred up, by the decided improvement and revival in dental literature, and believing that the opinion of my more modest brethren have been feebly expressed in this. The fact is, I am a modest man myself but could not hold in any longer, when so much is being done for the general good.

Yours, S. E. Harryman, D. D. S.

